# Durability of first antiretroviral treatment in HIV chronically infected patients: why change and what are the outcomes?

**DOI:** 10.7448/IAS.17.4.19797

**Published:** 2014-11-02

**Authors:** Patricia Moniz, Filipa Alçada, Susana Peres, Fernando Borges, Teresa Baptista, Ana Claudia Miranda, Isabel Antunes, Isabel Aldir, Fernando Ventura, Jaime Nina, Kamal Mansinho

**Affiliations:** Infectious Diseases and Tropical Medicine, Hospital Egas Moniz – Centro Hospitalar de Lisboa Ocidental, Lisbon, Portugal

## Abstract

**Introduction:**

First antiretroviral therapy (ART) is often switched to simpler, more potent or better tolerated regimens [1, 2]. Although discontinuation rates are frequently studied, the durability of regimens is rarely approached.

**Materials and Methods:**

Retrospective study with the following objectives: analyze first ART schemes and their durability in naive patients with chronic HIV-1 and 2 infections, evaluate factors influencing ART change, second-line ART and consequent virologic and immunologic responses. Patients had follow-ups in a Central University Hospital, started ART between January 2007 and December 2012 and changed first regimens. Clinical data was obtained from medical records and analyzed using the Statistical Package for the Social Sciences (version 20).

**Results:**

Of the 652 naive patients who started ART, 164 changed regimens. The majority had HIV-1 infection (n=158). The mean age was 43.9 years (standard deviation±14.3), with a male predominance of 57.9%. Regimens with efavirenz were the most common amongst HIV-1 patients (50%) followed by lopinavir/r (22%). In HIV-2 patients, lopinavir/r (n=3) regimens were most prevalent. First ART regimens had a mean duration of 12.1 months. There was no difference between NNRTI (59.8%) and protease inhibitor (40.2%) schemes regarding durability. Adverse reactions were the major cause of ART switching (55.5%) followed by therapy resistance (12.1%). Age was inversely related to durability (p=0.007 Mann-Whitney, Phi coefficient −0.161) and associated with the appearance of adverse reactions (p=0.04, Chi-square). Younger patients had a reduced risk of adverse reactions by 27%. Adverse reactions increased the risk of inferior durability by 40%. Psychiatric symptoms (28.4%) were the most prevalent, all attributed to efavirenz. The year of ART initiation was associated with different durability rates (p=0.005, Mann-Whitney). Patients started on ART before the year 2010 reduced the probability of inferior ART duration by 25.8%. After second-line ART regimens, TCD4+ counts>500 cell/µL were increased by 38% and favourable virologic outcome achieved in 84%.

**Conclusions:**

Adverse reactions were the main cause for ART switching, supporting a cautious approach when initiating regimens, particularly in older patients. All ART naive patients who changed initial therapy had favourable immunological and virologic responses.
